# Barriers to Effective Implementation of Mental Health Policies in India: A Comprehensive Analysis of Challenges and Solutions

**DOI:** 10.1192/j.eurpsy.2025.1657

**Published:** 2025-08-26

**Authors:** N. Yadav

**Affiliations:** 1 Psychiatry Social Work, Institute of Human Behaviour and Allied Sciences, Delhi, India

## Abstract

**Introduction:**

India is facing a growing mental health burden, with significant disparities in access to services despite the introduction of the National Mental Health Program (NMHP) and the Mental Healthcare Act (MHA). While these policies aim to improve mental health care, their implementation remains inconsistent due to a range of challenges. Understanding these barriers is crucial to enhancing mental health services across the country.

**Objectives:**

This study aims to identify the key challenges impeding the effective implementation of existing mental health policies in India, with a focus on rural-urban disparities, resource constraints, and sociocultural factors.

**Methods:**

A comprehensive literature review was conducted, examining peer-reviewed articles from databases such as PubMed, Scopus, and WHO policy documents, and reports from government and non-governmental organizations. Data was analysed to assess the primary obstacles related to funding, workforce shortages, stigma, policy integration, and infrastructure issues. Qualitative insights from key stakeholders in mental health services were also included.

**Results:**

The review revealed five primary challenges. Firstly, the insufficient financial allocation for mental health programs, leading to limited-service availability; secondly, a shortage of trained mental health professionals, particularly in rural areas; thirdly, a pervasive stigma surrounding mental health, hindering service uptake; fourthly, the poor integration of mental health care into primary health systems; and lastly, the bureaucratic inefficiencies and lack of infrastructure. These challenges disproportionately affect rural populations, exacerbating the urban-rural divide in mental health care delivery.

**Conclusions:**

The effective implementation of mental health policies in India is undermined by systemic challenges such as inadequate resources, workforce gaps, and sociocultural barriers. Addressing these issues requires targeted interventions, including increased investment in mental health services, enhanced training programs, stigma reduction campaigns, and better integration of mental health care into general healthcare frameworks. A coordinated, multi-level approach is essential to overcoming these barriers and achieving meaningful improvements in mental health outcomes across India.

**Disclosure of Interest:**

None Declared

